# Long-term outcomes of a case-control lung transplant cohort after SARS-CoV-2 infection

**DOI:** 10.3389/frtra.2025.1583919

**Published:** 2025-05-21

**Authors:** Sandrine Hanna, Rami Hallak, Susanna M. Leonard, Samantha Morrison, Sarah Peskoe, Jordan Whitson, John M. Reynolds, Cameron R. Wolfe, Hakim Azfar Ali

**Affiliations:** ^1^ Division of Pulmonary, Allergy and Critical Care, Department of Medicine, Duke University Hospital, Durham, NC, United States; ^2^ Department of Pulmonary Critical Care, Indiana University, Indianapolis, IN, United States; ^3^ Department of Medicine, Baptist Health Paducah, Paducah, KY, United States; ^4^ Division of Pulmonary, Allergy and Critical Care, Department of Medicine, University of Pennsylvania, Philadelphia, PA, United States; ^5^ Department of Biostatistics and Bioinformatics, Duke University School of Medicine, Durham, NC, United States; ^6^ Division of Infectious Diseases, Department of Medicine, Duke University Hospital, Durham, NC, United States

**Keywords:** COVID-19, SARS-CoV-2 infection, acute cellular rejection (ACR), lung transplant, immune compromise, FEV 1, mortality

## Abstract

**Background:**

Respiratory viruses can impact the allograft function in lung transplant recipients, but it is unknown if this occurs with SARS-CoV-2 infection. We studied the long-term outcomes of lung transplant recipients infected with SARS-CoV-2.

**Methods:**

This single-center retrospective study compared lung transplant recipients with SARS-CoV-2 between June 2020 and April 2021 with a matched control group. Within the SARS-CoV-2 cohort, univariable associations between clinical factors and outcomes were tested. Changes in pulmonary function tests were analyzed. Primary endpoints included acute cellular rejection and all-cause mortality within 12 months.

**Results:**

Fifty-three lung transplant recipients were infected with SARS-CoV-2. The median age was 64 years. 29 (54.7%) were managed outpatient, and 24 (45.3%) required hospitalization, with 13 intensive care unit admissions. All-cause mortality was 24.5%. Within the SARS-CoV-2 cohort, older age was significantly associated with all-cause mortality (*p*-value 0.017) as was ICU admission (*p* = 0.009) and an A1C > 6.5 (*p* = 0.033). The mean change in FEV1 was −1.1% at 3 months with minimal change at 6 and 12 months (−2.6% and −1% respectively), all compared to baseline. Acute cellular rejection was identified in 13.7% of the SARS-CoV-2 cohort compared to 11.8% in the matched control group; it was not significantly associated with the infection status (*p* = 0.706). However, all-cause mortality was significantly associated with infection status (*p* = 0.019).

**Conclusion:**

Long-term outcomes of SARS-CoV-2 in lung transplant recipients are widely variable. Within the SARS-CoV-2 cohort, all-cause mortality was 24.5%, and older age was significantly associated with mortality. We did not observe significant declines in FEV1 in this group.

## Introduction

The COVID-19 pandemic, due to the SARS-CoV-2 virus, represents an ongoing global health burden resulting in considerable morbidity and mortality. The SARS-CoV-2 virus primarily targets the lungs, where it infiltrates epithelial cells, facilitated by the spike glycoprotein on its outer envelope, resulting in the up-regulation of various inflammatory cytokines into the circulation and potentially leading to an acute respiratory syndrome in severe cases (1). As SARS-CoV-2 has evolved, newer variants have dominated in different eras, each with variable infectivity, immune evasion, and disease severity (2).

Comorbidities, particularly immunosuppression, significantly affect SARS-CoV-2 by increasing infectivity, modifying disease progression, and potentially attenuating vaccine response (3). Consequently, solid organ transplant recipients (SOTRs), due to their chronic immunosuppression and medical comorbidities, have a high early post-infectious mortality rate, ranging from 13% to 30% in different published cohorts (4–7). Other viruses have been shown to impact long-term rejection risk in lung transplant recipients. Whether this occurs in the aftermath of SARS-CoV-2 infection is not yet known.

This study aims to describe our experience with SARS-CoV-2 infection in LTRs, specifically the long-term follow-up of these patients up to 1 year after infection. We studied the impact on performance status via the change in the forced expiratory volume in the first second (FEV1) post-infection in LTRs. We compared the rates of acute cellular rejection (ACR) and mortality post-infection in LTRs to those in a matched control group.

## Methods

This retrospective observational study was conducted at a large-volume tertiary medical center by reviewing LTRs' medical charts. In our center, we follow transplant patients serially as per our transplant protocol. This includes updating records in the patient's chart following outpatient visits, pulmonary rehabilitation visits, lab workup visits, procedural visits, and telehealth visits.

### Study populations

We retrospectively reviewed our patients' medical charts between June 1st, 2020, and April 30th, 2021, to identify those infected with SARS-CoV-2. Infection was confirmed by either a polymerase chain reaction (PCR) test or a rapid antigen test performed at home or at a healthcare facility. Demographic data including age, sex, BMI, primary disease, type of transplant, date of transplant, and presence of chronic kidney disease were obtained for each patient. Additionally, we recorded the immunosuppression regimen, any augmented treatments in the preceding 2 months, baseline FEV1, and vaccination status. We collected data on each patient's condition during the SARS-CoV-2 infection—particularly regarding hospitalization, the severity of the disease, treatment received, oxygen and ventilator/support need, and mortality. By hospital protocol, the third immunosuppressive agent was held during the infection. We continued to track patients who survived post-infection for any recorded FEV1 and transbronchial lung biopsies (TBLB) at 3, 6, and up to 12 months post-SARS-CoV-2 infection.

We included a control group of LTRs without SARs-CoV-2 to compare ACR and all-cause mortality rates to the study cohort. The control group was comprised of patients who did not have SARS-CoV-2 infection from a concomitant study using 1:1 matching based on age at transplant (±5 years), the number of years since transplant (0–2, 2–4, 4–6, 6+), and transplant type (bilateral vs. single). The index date for the control group was 12 months before the last recorded biopsy (April 7, 2022), and the index date for the SARS-CoV-2 group was the date of the SARS-CoV-2 infection. Two patients from the SARS-CoV-2 group were not matched to any patient in the control group and were excluded from the subsequent analysis.

Our study was approved by the institutional review boards (IRB) at our hospital (Pro00105791).

### Study endpoints

Primary endpoints for the matched analysis were ACR and all-cause mortality within 12 months. ACR was defined as any TBLB performed within 12 months from the index date that resulted in an A1 stage rejection or higher, as defined by ISHLT guidelines. For the SARS-CoV-2 cohort, pre-infection baseline FEV1 and follow-ups at approximately 3, 6, and 12 months were recorded. Baseline FEV1 was defined as the most recent measure recorded before infection. Percent change in FEV1 compared to baseline was calculated.

### Statistical analysis

We provide descriptive statistics tables for the SARS-CoV-2 patients by hospitalization status and all-cause mortality. We tested if various clinical covariates differed by these outcomes using the Wilcoxon Rank Sum Test and Fisher's Exact Test. To compare rates of ACR between the SARS-CoV-2 cohort and the control cohort, we modeled the odds of ACR by SARS-CoV-2 status using a conditional logistic regression model stratifying on the matched pairs. We presented the odds ratio (OR), 95% confidence interval, and *p*-value. To compare all-cause mortality within 12 months between the 2 cohorts, we modeled the odds of mortality by SARS-CoV-2 status using a conditional logistic regression model stratifying on the matched pairs and presented the odds ratio, 95% confidence interval, and *p*-value.

All of the surviving SARS-CoV-2 patients were presented with changes in FEV1 at approximately 3, 6, and 12 months compared to baseline. Change in FEV1 was defined as (FEV1 follow-up—Baseline)/Baseline. To estimate the change in FEV1 over time, we fit a linear mixed effect model to time, including a random intercept for the patient, and presented the estimate, 95% CI, and *p*-value.

## Results

### Demographics

Fifty-three LTRs were confirmed to have SARS-CoV-2 infection between June 1, 2020, and April 30, 2021. The median age was 64 years, ranging between 21 and 81 years 58.5% of our population were male. Most of our LTRs (92.5%) had bilateral orthotopic lung transplants (BOLT). Most of our patients were of normal weight (32.1%) or overweight (45.3%) and had an eGFR < 60 (81.1%), with a median eGFR of 42. Thirty-nine (73.6%) of our patients were on three immunosuppressive agents at baseline, with the remainder on two agents. Four patients received augmented immunosuppression 2 months before infection, and four patients had previously received alemtuzumab. Most of our patients were unvaccinated at the time of infection (86.8%) (Tables 1, 2).

**Table 1:** SARS-CoV-2 patient demographics by hospitalization status.

Characteristic	Hospitalized (*N* = 24)	Not hospitalized (*N* = 29)	Overall (*N* = 53)	*p*-value*
Age (years)	64 [55, 68.5]	63 [53, 70]	64 [53, 70]	0.858
Age at 1st transplant (years)	59 [50.5, 63]	57 [37, 63]	58 [48, 63]	0.405
Sex				1
Female	10 (41.7%)	12 (41.4%)	22 (41.5%)	
Male	14 (58.3%)	17 (58.6%)	31 (58.5%)	
BMI (kg/m^2^)	26.2 [22.5, 27.5]	25.9 [24, 29.7]	26.1 [23.4, 29.7]	0.844
BMI categories				0.875
Underweight	0 (0%)	1 (3.4%)	1 (1.9%)	
Normal weight	9 (37.5%)	8 (27.6%)	17 (32.1%)	
Overweight	10 (41.7%)	14 (48.3%)	24 (45.3%)	
Obese	5 (20.8%)	6 (20.7%)	11 (20.8%)	
CKD (eGFR < 60)	21 (87.5%)	22 (75.9%)	43 (81.1%)	0.318
eGFR (ml/min)	39.5 [29.5, 51]	49 [34, 57]	42 [32.4, 55]	0.148
HBA1C ≥ 6.5 (*n* = 52)				0.061
*N*	23	29	52	
Yes	7 (30.4%)	2 (6.9%)	9 (17.3%)	
Type of Transplant				0.617
BOLT	23 (95.8%)	26 (89.7%)	49 (92.5%)	
SOLT	1 (4.2%)	3 (10.3%)	4 (7.5%)	
Immunosuppression Regimen				1
Two agents: CI + steroids	6 (25%)	8 (27.6%)	14 (26.4%)	
Three agents: CI + steroids	18 (75%)	21 (72.4%)	39 (73.6%)	
Augmented treatment (*n* = 4)				0.500
*N*	3	1	4	
Pulse Steroids + taper	1 (33.3%)	0 (0%)	1 (25%)	
Antithymoglobulin	0 (0%)	1 (100%)	1 (25%)	
Others (Rituximab for AMR)	2 (66.7%)	0 (0%)	2 (50%)	
Received Alemtuzumab	3 (12.5%)	1 (3.4%)	4 (7.5%)	0.318
Covid Vaccination Status				0.482
Non-vaccinated	20 (83.3%)	26 (89.7%)	46 (86.8%)	
mRNA vaccine one dose	1 (4.2%)	2 (6.9%)	3 (5.7%)	
mRNA vaccine two doses	3 (12.5%)	1 (3.4%)	4 (7.5%)	
Hospital LOS (days) (*n* = 24)	10 [6.8, 17.8]	-	-	-
Admitted to ICU (*n* = 53)	13 (54.2%)	-	-	-
ICU LOS (days) (*n* = 13)	14 [7, 22]	-	-	-
Oxygen/Ventilator Needs (*n* = 53)				<0.001
None	4 (16.7%)	28 (96.6%)	32 (60.4%)	
Low flow Nasal Cannula	10 (41.7%)	0 (0%)	10 (18.9%)	
HFNC	2 (8.3%)	0 (0%)	2 (3.8%)	
Mechanical Ventilation	6 (25%)	0 (0%)	6 (11.3%)	
Mechanical Ventilation and ECMO	2 (8.3%)	0 (0%)	2 (3.8%)	
Same home baseline O_2_	0 (0%)	1 (3.4%)	1 (1.9%)	
SARS-CoV-2 Treatments Received				
Dexamethasone	21 (87.5%)	3 (10.3%)	24 (45.3%)	<0.001
Remdesivir	20 (83.3%)	0 (0%)	20 (37.7%)	<0.001
Monoclonal Antibodies	1 (4.2%)	12 (41.4%)	13 (24.5%)	0.003
Convalescent Plasma	3 (12.5%)	0 (0%)	3 (5.7%)	0.086
All-cause Mortality^a^	9 (37.5%)	4 (13.8%)	13 (24.5%)	0.060

Median [Q1, Q3] and *n* (%) are presented.

^a^ 8 of the mortalities were due to SARS-CoV-2 or related complications.

* *p*-values from Wilcoxon Rank Sum Test and Fisher's Exact Tests.

**Table 2:** SARS-CoV-2 patient demographics by all-cause mortality within 12 months.

Characteristic	Died within 12 months (*N* = 13)	Survived (*N* = 40)	Overall (*N* = 53)	*p*-value*
Age (years)	67 [64, 70]	58 [47.2, 68]	64 [53, 70]	0.017
Age at 1st transplant (years)	62 [60, 63]	53.5 [36.8, 62.2]	58 [48, 63]	0.020
Sex				1
Female	5 (38.5%)	17 (42.5%)	22 (41.5%)	
Male	8 (61.5%)	23 (57.5%)	31 (58.5%)	
BMI (kg/m^2^)	26.6 [23.7, 27.4]	25.9 [23.2, 29.8]	26.1 [23.4, 29.7]	0.975
BMI categories				0.509
Underweight	0 (0%)	1 (2.5%)	1 (1.9%)	
Normal weight	4 (30.8%)	13 (32.5%)	17 (32.1%)	
Overweight	8 (61.5%)	16 (40%)	24 (45.3%)	
Obese	1 (7.7%)	10 (25%)	11 (20.8%)	
CKD (eGFR < 60)	12 (92.3%)	31 (77.5%)	43 (81.1%)	0.419
eGFR (ml/min)	39 [29, 51]	43.5 [34.8, 55.2]	42 [32.4, 55]	0.380
HBA1C ≥ 6.5 (*n* = 52)				0.033
*N*	13	39	52	
Yes	5 (38.5%)	4 (10.3%)	9 (17.3%)	
Type of transplant				0.561
BOLT	13 (100%)	36 (90%)	49 (92.5%)	
SOLT	0 (0%)	4 (10%)	4 (7.5%)	
Immunosuppression Regimen				0.292
Two agents: CI + steroids	5 (38.5%)	9 (22.5%)	14 (26.4%)	
Three agents: CI + steroids	8 (61.5%)	31 (77.5%)	39 (73.6%)	
Augmented treatment (*n* = 4)				1
*N*	2	2	4	
Pulse Steroids + taper	0 (0%)	1 (50%)	1 (25%)	
Anti-Thymocyte Globulin	1 (50%)	0 (0%)	1 (25%)	
Others (Rituximab for AMR)	1 (50%)	1 (50%)	2 (50%)	
Received Alemtuzumab	3 (23.1%)	1 (2.5%)	4 (7.5%)	0.042
Covid Vaccinated Status				0.486
Non-vaccinated	13 (100%)	33 (82.5%)	46 (86.8%)	
mRNA vaccine one dose	0 (0%)	3 (7.5%)	3 (5.7%)	
mRNA vaccine two doses	0 (0%)	4 (10%)	4 (7.5%)	
Hospitalized	9 (69.2%)	15 (37.5%)	24 (45.3%)	0.060
Hospital LOS (days)				0.929
*N*	9	15	24	
Median [Q1, Q3]	10 [7, 17]	11 [5.5, 17.5]	10 [6.8, 17.8]	
Admitted to ICU	7 (53.8%)	6 (15%)	13 (24.5%)	0.009
ICU LOS (days)				0.616
*N*	7	6	13	
Median [Q1, Q3]	10 [6, 19.5]	14 [11, 36.5]	14 [7, 22]	
Oxygen/Ventilator Needs				<0.001
None	3 (23.1%)	29 (72.5%)	32 (60.4%)	
Low flow Nasal Cannula	2 (15.4%)	8 (20%)	10 (18.9%)	
HFNC	1 (7.7%)	1 (2.5%)	2 (3.8%)	
Mechanical Ventilation	4 (30.8%)	2 (5%)	6 (11.3%)	
Mechanical Ventilation and ECMO	2 (15.4%)	0 (0%)	2 (3.8%)	
Same home baseline O_2_	1 (7.7%)	0 (0%)	1 (1.9%)	
SARS-CoV-2 treatments received				
Dexamethasone	10 (76.9%)	14 (35%)	24 (45.3%)	0.011
Remdesivir	9 (69.2%)	11 (27.5%)	20 (37.7%)	0.010
Monoclonal Antibodies	1 (7.7%)	12 (30%)	13 (24.5%)	0.148
Convalescent Plasma	2 (15.4%)	1 (2.5%)	3 (5.7%)	0.145

Median [Q1, Q3] and *n* (%) are presented.

* *p*-values from Wilcoxon Rank Sum Test and Fisher's Exact Tests.

### Outcomes in the SARS-CoV-2 cohort

Twenty-nine (54.7%) LTRs did not require hospitalization, and those patients did not have new oxygen requirements, while twenty-four (45.3%) required hospitalization, with a mean length of hospital stay of 20.6 days. Thirteen patients (24.5%) required admission to the ICU—of those in the ICU, 8 patients required mechanical ventilation, 2 of whom required ECMO. The majority of the hospitalized patients were treated with dexamethasone (87.5%) and remdesivir (83.3%), while most of the non-hospitalized patients did not require treatment (3 patients received dexamethasone, and 12 patients received monoclonal antibody infusions). Overall, 40 (75.5%) LTRs survived at least 12 months post-SARS-CoV-2 infection. Thirteen (24.5%) died within 12 months due to any cause, 8 of whom died due to the SARS-CoV-2 infection and its complications (Tables 1, 2). The remaining deaths not related to COVID-19 were attributed to metastatic cancer (1 patient), non-COVID-related infections during separate admissions (3 patients), and chronic lung allograft dysfunction (CLAD, 1 patient).

The patient's age during infection was significantly associated with all-cause mortality (*p*-value 0.017). Those who survived were younger, with a mean age of 55.9 years, compared to those who died, with a mean age of 67.6 years. Having an HbA1C ≥ 6.5, previous administration of alemtuzumab, and being admitted to the ICU were statistically significantly associated with mortality with *p*-values of 0.033, 0.042, and 0.009, respectively, although the event rates were small (Table 2). Associations between the outcomes (hospitalization, mortality) and oxygen needs and SARS-CoV-2 treatments were not interpreted due to confounding resulting from variations in access to treatments and the severity of the disease.

### ACR and all-cause mortality: matched analysis

Matching between our SARS-CoV-2 LTRs and our non-infected control cohort LTRs resulted in 51 matched patients per cohort (Table 3). 13.7% of the SARS-CoV-2 patients suffered ACR within 12 months post-infection compared to 11.8% in the control group. Both groups' biopsy frequency differences can be noted in Table 3. 21 (47.7%) of the SARS-COV-2 population did not have biopsies vs. 29 (64.4%) of the control cohort. The association between ACR within 12 months and SARS-CoV-2 infection was not statistically significant, with an odds ratio of 1.333 (0.298, 5.957) and a *p*-value of 0.706. Infection with SARS-CoV-2 in LTRs was significantly associated with increased odds of 12-month all-cause mortality (OR 6.00, 95% CI 1.343–26.81, *p*-value 0.019) (Table 4).

**Table 3:** Demographics, rates of ACR, and mortality for matched SARS-CoV-2 cohort and control cohort.

Characteristics	SARS-CoV-2 patients (*N* = 51)	Control cohort (*N* = 51)
Age at transplant (years)		
Mean (SD)	53.3 (14.9)	53.6 (14.4)
Median [Q1, Q3]	58 [48.5, 63]	57 [48.5, 64]
Range	(14, 74)	(16, 72)
Years since transplant (from index date)^a^		
0–2	17 (33.3%)	17 (33.3%)
2–4	7 (13.7%)	7 (13.7%)
4–6	13 (25.5%)	13 (25.5%)
6+	14 (27.5%)	14 (27.5%)
Sex		
Male	29 (56.9%)	28 (54.9%)
Female	22 (43.1%)	23 (45.1%)
Transplant Type		
BOLT	49 (96.1%)	49 (96.1%)
SOLT	2 (3.9%)	2 (3.9%)
Acute cellular rejection^b^	7 (13.7%)	6 (11.8%)
No Acute cellular rejection	44 (86.3%)	45 (88.2%)
Patients with no biopsies	21 (47.7%)	29 (64.4%)
Patients with at least one biopsy but no rejection	23 (52.3%)	16 (35.6%)
All-cause 12 month mortality	13 (25.5%)	3 (5.9%)
Time index to death (days)		
*N*	13	3
Mean (SD)	97.6 (107.8)	330 (9.8)
Median [Q1, Q3]	43 [7, 144]	333 [326, 335.5]
Range	(5, 309)	(319, 338)

^a^ Index date for covid patients was the date of infection, for control patients was April 7, 2020.

^b^ At least one biopsy of Grade A1 or higher within 12 months. Patients with no biopsies recorded were considered negative for ACR.

**Table 4:** Separate conditional logistic regressions testing the association between COVID status and outcomes acute cellular rejection and all-cause mortality within 12 months (*N* = 102).

Outcome	Covariate	OR	95% C.I.	*p*-value*
Acute cellular rejection^a^	Covid status (Yes)	1.333	(0.298, 5.957)	0.706
All-cause mortality	Covid status (Yes)	6.00	(1.343, 26.81)	0.019

^a^As defined by a biopsy of grade A1+.

* Wald Test.

### Change in FEV1 in the SARS-CoV-2 cohort

**Figure 1:**
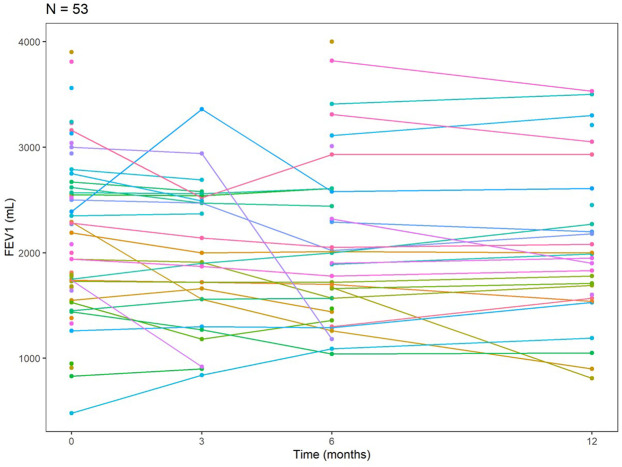
The trajectory of FEV1 at baseline, 3, 6, and 12 months for *N* = 53 SARS-CoV-2 patients.

Among our LTRs with SARS-CoV-2 infection (*n* = 53), 27 performed pulmonary function tests (PFTs) at approximately 3 months post-infection, 37 patients at approximately 6 months, and 28 patients at approximately 12-month intervals. The mean change in FEV1 relative to baseline was −1.1% at around 3 months post-infection, −2.6% around 6 months, and −1% at about 12 months (Table 5). The trajectory of FEV1 for the SARS-CoV-2 patients at baseline, about 3, 6, and 12 months, is shown in a spaghetti plot (Figure 1). From the linear mixed effects model, for each additional month past the baseline, FEV1 decreases by 10.14 ml on average; however, this finding was not statistically significant [estimate: −10.14 ml (−21.36, 1.09), *p*-value 0.076].

**Table 5:** Change in FEV1 for the SARS-CoV-2 cohort (liters).

FEV1 change	SARS-CoV-2 patients (*N* = 53)
Change in FEV1 3 months	
*N*	27
Mean (SD)	−0.011 (0.216)
Median [Q1, Q3]	−0.015 [−0.074, 0.02]
Range	(−0.471, 0.75)
Change in FEV1 6 months	
*N*	37
Mean (SD)	−0.026 (0.283)
Median [Q1, Q3]	−0.067 [−0.111, 0.024]
Range	(−0.607, 1.271)
Change in FEV1 12 months	
*N*	28
Mean (SD)	−0.01 (0.347)
Median [Q1, Q3]	−0.064 [−0.095, 0.052]
Range	(−0.607, 1.479)

Change in FEV1 = (FEV1 follow up—Baseline)/Baseline.

## Discussion

The temporal scope of our analysis corresponds to the relatively short period since the onset of the COVID-19 pandemic. Data on SARS-CoV-2 infection in long-term recipients (LTRs) remain insufficient, particularly regarding its long-term outcomes and recovery. Our study adds to the growing body of knowledge on COVID-19 within the transplant population, particularly emphasizing the diverse genetic strains and their potential variability in influencing outcomes. Notably, our study is unique in that most patients were unvaccinated during the early stages of the pandemic. We included a 12-month post-infection follow-up and used a matched case-control design to evaluate the rates of ACR and all-cause mortality. Our cohort was infected during a timeframe when the predominant strains were the alpha, beta, gamma, and delta variants of the virus.

Overall, the results of our study reveal that the outcome of SARs-CoV-2 infection in LTRs is widely variable. This was, likewise, presented in other reported studies. A study from Sweden that included 47 LTRs infected with SARS-CoV-2 revealed a mortality rate of 4.5% and a severe disease course in those having low pre-infection lung function (8). Another study from France described SARS-CoV-2 infection outcomes in 35 LTRs, with 88.6% required hospitalization and a 14.3% mortality rate (9). Mortality was greater in a multicenter study of 44 LTRs from Spain (39%) (10). Likewise, the severity of the disease was reflected in a single-center study from New York evaluating 32 LTRs infected with SARS-CoV-2, revealing a mortality rate of 34% (11). Our study continues to reflect these variable outcomes, where the all-cause mortality rate within 12 months post-infection was 24.5%, and the SARS-CoV-2-specific mortality rate was 15.1%. From the matched analysis, the odds of all-cause mortality for infected LTRs were notably six times the odds for non-infected LTRs, reflecting the pathogenicity of the virus.

Our study noted increasing age as a significant risk factor for all-cause mortality from SARS-CoV-2, as noted elsewhere (8, 12, 13). Similarly, a study of COVID-19 cases from November 2020 to February 2021 reported an overall 90-day all-cause mortality rate of 17%, with a higher mortality among patients with severe disease (54%) (14). Conversely, other demographic factors and immunosuppressive regimens did not appear to be associated with mortality. In our study, HbA1c ≥ 6.5, previous receipt of alemtuzumab, and ICU admission were significantly associated with mortality. Similar results indicating that elevated HbA1C increases mortality risk were found in a systemic review and meta-analysis that studied the general population (15). Alemtuzumab has not previously been reported to be associated with increased COVID-19–related mortality in the transplant population. The observed association in our review may not accurately reflect the broader population due to the limited number of patients included. Nonetheless, given its profound lymphocyte-depleting effects, alemtuzumab is theoretically considered to increase susceptibility to infections. Most of the available data on COVID-19 mortality and Alemtuzumab originates from studies involving multiple sclerosis patients, with limited literature on lung transplant recipients (16, 17).

Most of our patients were not vaccinated, reflecting enrollment in our study and vaccine availability at the time. Vaccination against the SARS-CoV-2 virus was launched in December 2020 in the United States (18), suggesting that some of our patients may have acquired infection in the pre-vaccination era. Truly, our group represents LTRs who were infected with the early variants of the SARS-COV-2 virus, a timeline when vaccinations were newly introduced and perhaps still not widely acknowledged. Despite the majority being unvaccinated, more than half of our patients did not require hospitalization or oxygen needs.

Respiratory infections, the majority of the post-transplant insults, have been perceived to affect the allograft function by causing acute cellular rejection (19, 20). However, the clinical predictors of developing acute rejection post-infection remain lacking. Some studies revealed an increased risk of BOS following respiratory infections (21–23). Our study has shown that the rate of ACR in LTRs who acquired the SARS-CoV-2 virus and LTRs without infection was remarkably similar, suggesting post-viral ACR does not always occur. However, we note that patients who died within the 12 months did not have the same opportunity to develop ACR, therefore, future studies may want to consider semi-competing risks of ACR and death in this population.

Spirometry is considered a clinical marker of any respiratory insult in lung transplants. Any significant decline in FEV1 and FVC is often associated with infection or allograft rejection (24, 25). We expected to observe a substantial drop in the baseline FEV1 of our patients following infection with the SARS-CoV-2 virus, however, that was not the case in our cohort. Our findings showed that the mean change in FEV1 was −1.1% at about 3 months post-infection, −2.6% at about 6 months, and −0.01 at about 12 months, and time was not found to be significantly associated with FEV1. However, we observed stabilization after an early decline in FEV1 in the 3 months post-infection (Figure 1). This remains a descriptive interpretation and is limited by various missing time points.

This study was limited by the retrospective nature of the observational chart review and some missing data points, especially regarding PFTs and biopsy-proven ACR. We also did not have imaging data for more than half of the patients, primarily because many did not have inpatient care. Additionally, only a limited number underwent biopsies in this cohort, reflecting the reduced frequency of surveillance biopsies related to COVID-related logistical constraints. As a result, the rates of acute cellular rejection (ACR) may have been underestimated. Although our infected cohort is larger than other reported studies, the numbers remain small. Based on available data, follow-up at 3-, 6-, and 12-month intervals varied in timing between patients. Finally, as previously noted, our cohort of lung transplant recipients with SARS-CoV-2 infection was unvaccinated, which limits the generalizability of our findings to the post-vaccination era, particularly in the context of emerging variants beyond the early SARS-CoV-2 strains.

## Conclusion

The outcome of SARS-CoV-2 infection in LTRs is widely variable. Our study described the outcome and is one of the few with data on long-term follow-up of patients who acquired the infection, especially focusing on an early strain of the virus. The SARS-CoV2-specific mortality of 15.1% and a high all-cause mortality rate of 24.5% show the vulnerability of this population to novel infections with indirect downstream consequences leading to high morbidity and mortality. Older age is significantly associated with mortality. Although the numbers were small, higher mortality was seen in patients requiring ICU admission, diabetics with higher A1C, and patients receiving alemtuzumab, providing hypotheses for further study in this population. The majority of our patients were unvaccinated or perhaps in the pre-vaccination era. Significantly higher rates of ACR or decline in FEV1 were not observed with the SARS-CoV-2 infection. The long-term impact on chronic allograft function remains to be seen. Given the emergence of subsequent strains of the virus, our findings establish a baseline for comparing the impact of these later variants on the lung transplant population, particularly in the post-vaccination era.

## Data Availability

The original contributions presented in the study are included in the article/Supplementary Material, further inquiries can be directed to the corresponding authors.
